# Exploring the self-efficacy of patients with diabetes: its role as a predictor of diabetes management and well-being

**DOI:** 10.3389/fendo.2024.1347396

**Published:** 2024-05-21

**Authors:** Ayoub Ali Alshaikh, Faisal Saeed Al-Qahtani, Saif Aboud M. Alqahtani, Ahmad Ali AlFarhan, Ali Mushabbab Al Nuwayhidh, Ayman Mohammed Madkhali, Riyad Saeed AlQahtani, Ali Fayez AlAsmari, Abdulaziz Saeed Alserhani, Hatim Ahmed Alqubaisi, Ziyad Saad Saeed Aldawh, Abdulmalik Khalid Al Bin Ahmad, Ramy Mohamed Ghazy

**Affiliations:** ^1^ Family and Community Medicine Department, College of Medicine, King Khalid University, Abha, Saudi Arabia; ^2^ Internal Medicine Department, College of Medicine, King Khalid University, Abha, Saudi Arabia; ^3^ Medical Colleague, College of Medicine, King Khalid University, Abha, Saudi Arabia; ^4^ Clinical pharmacist Intern, College of Pharmacy, King Khalid University, Abha, Saudi Arabia; ^5^ Tropical Health Department, High Institute of Public Health, Alexandria University, Alexandria, Egypt

**Keywords:** well-being, self-efficacy, diabetes mellitus, Saudi Arabia, diabetes control

## Abstract

**Background:**

Self-efficacy is a popular psychological concept that refers to an individual’s perception or belief in his ability to perform specific actions. This study aimed to assess the predictive value of self-efficacy, measured using the Self-Efficacy for Managing Chronic Disease 6-Item Scale (SEM6S) questionnaire, for diabetes management and overall well-being in patients with diabetes.

**Subject and methods:**

An anonymous online cross-sectional study was conducted to evaluate the self-efficacy of diabetic patients in the Asser region of Saudi Arabia. The participants were requested to upload their most recent glycated hemoglobin A1C (HbA1C) measurements taken in the last three months, which helped in the accurate categorization of their diabetes as either controlled or uncontrolled. We used the valid Arabic version of the SEM6S and WHO-5 well-being questionnaires to assess patient self-efficacy and well-being.

**Results:**

A cohort of 342 patients was enrolled in the study, 67.25% were married, their mean age was 43.17 ± 17.61 years, and 52.69% had university-level or higher education. Among the participants, 46.0% exhibited well-being, while 24.9% reported poor well-being, including 9.4% who were identified as experiencing depression. The mean scores of self-efficacy and well-being were significantly higher among those with controlled diabetes versus uncontrolled diabetes (40.86 ± 13.26 vs. 36.48 ± 13.26) and (67.35 ± 21.22 vs. 60.93 ± 25.05), respectively. The predictors of glycemic control were self-efficacy [Odds ratio (OR)=1.03 (95%CI, 1.01-1.06, *P*=0.002], having other chronic diseases [OR=3.25 (95%CI), *P*<0.001], having type 1 diabetes [OR=7.16, 95%CI, *P*=0.005], being Saudi [OR=7.67, (95%CI, *P*=0.027], working in a public sector [OR=0.15, (95%CI, 0.05-0.44), *P*=0.005], being unemployed [OR=0.19, (95%CI, 0.06-0.59), *P*=0.005], being a smoker [OR=0.44, 95%CI, 0.19-0.98, *P*=0.048], and duration of diabetes between 6-10 years [OR= 0.33, 95%CI, 0.11-0.95), *P*=0.043] or more than 10 years OR=0.32, 95%CI, 0.12-0.86), *P*=0.026]. The main determinants of well-being were having self-efficacy [OR=1.07 (95%CI, 1.04-1.09), *P* = 0.0001], having public health insurance [OR=4.36 (95%CI, *P*=0.015], and education level (read and write) [OR=0.13 (95%CI,.02-.70), *P*=0.021].

**Conclusions:**

The study reveals that non-modifiable and modifiable factors, including self-efficacy, play a crucial role in diabetes control. The study recommends providing targeted educational interventions, using different social media platforms, psychosocial support programs, and inclusive healthcare policies to improve diabetes control and mental well-being among diabetic patients.

## 1 Introduction

The prevalence of diabetes mellitus (DM) in Saudi Arabia ranks second highest in the Middle East and seventh worldwide. (1) It is estimated that over 7 million people are diabetic, with nearly 3 million having pre-diabetes. (1) In Saudi Arabia, a comprehensive epidemiological health study was conducted, focusing on adults aged 30 to 70 years who live in selected households. The survey found that diabetes was diagnosed in 4,004 of the 16,917 survey participants, accounting for approximately 23.7% of the population. (2) This substantial increase, exceeding ten times in the last three decades, has led to elevated rates of mortality and morbidity, contributing to compromised health and a reduced quality of life (QoL) (1).

**Figure 1:**
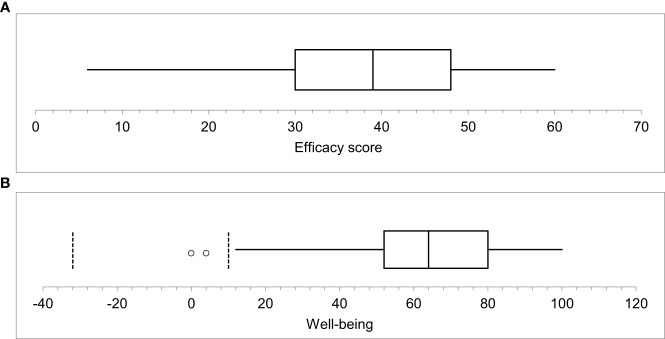
**(A, B)** show the summary statistics of the self-efficacy and wellbeing scores. The maximum score of the self-efficacy is 60, and the maximum score of wellbeing is 100. The mean of the efficacy score was 38.4 ± 13.3, the median was 390. Additionally, the wellbeing mean was 63 ± 23.1 and the median score was 64.0.

Self-efficacy is a popular psychology concept that refers to an individual’s perception or belief in his ability to perform specific actions. (3) Recognizing the importance of self-efficacy as a fundamental requirement for successful self-care of chronic diseases, there is growing recognition of its importance in managing DM. Self-care for diabetes encompasses a spectrum that spans from increased awareness of people living with DM to more engaged and proactive participation in the overall management process (4) Estimating self-efficacy for self-care routines in diabetic patients is a crucial step toward better diabetes control. According to self-efficacy theory, the effective completion of the action plan is more important than the plan itself. (5) Promoting a higher level of self-efficacy has been associated with improved DM self-care practices in nutrition, exercise, medication adherence, blood glucose testing, risk reduction behaviors (e.g., smoking cessation), and foot care, which leads to better glycemic control and QoL. (6) The Self-Efficacy for Managing Chronic Disease 6-Item Scale (SEM6S) is a validated, brief questionnaire designed for use in research and clinical settings. This simple assessment tool helps patients evaluate their confidence in managing chronic conditions. The questionnaire assesses one’s self-efficacy in dealing with fatigue, discomfort, pain, mental distress, and other symptoms associated with chronic disease treatment (7) **Figure 1**.

Patients with diabetes exhibit a higher prevalence of depression, estimated to be two to three times higher than that observed in the general population, thus negatively impacting both their QoL and diabetes-related outcomes. (8, 9) In light of this, routine screening for depression is recommended for diabetic patients. The World Health Organization-Five Well-Being Index (WHO-5) appears as a concise and positively framed instrument designed to evaluate emotional well-being over a 14-day span. Extensive research in adults, both with and without diabetes, using the Structured Clinical Interview for the Diagnostic and Statistical Manual (DSM)-IV as a benchmark, has demonstrated the WHO-5’s excellent sensitivity (94–100%) and specificity (78%). Due to its brevity and emphasis on positive affect, the WHO-5 holds promise as a suitable screening tool for assessing low emotional well-being and depressive tendencies in patients with diabetes (10, 11) **Figure 1**.

In this study, we hypothesize that higher levels of self-efficacy among patients with diabetes would be associated with better diabetes management and overall well-being. The main objective of this study was to explore the association between self-efficacy, assessed through the SEM6S questionnaire, and glycemic control in Saudi Arabian diabetic patients. Additionally, our focus expanded to evaluating the well-being of diabetic individuals and determining the role of self-efficacy as a predictor of overall well-being. This study may provide useful insights for healthcare interventions to improve the QoL for diabetic patients in Saudi Arabia.

## 2 Subject and method

### 2.1 Study setting and study population

An anonymous online cross-sectional study was used to assess self-efficacy and well-being among diabetic patients in the Asser region of Saudi Arabia. Participants were selected using convenience and snowball sampling methods. Using G*power software (version 3.1, Franz Faul, Universitat Kiel, Germany), the minimum required sample size was 210 (105 having glycated hemoglobin A1C (HbA1C) > 7%, and 105 having HbA1C ≤ 7%), presuming an effect size of 0.5, an alpha error of 5%, and a power of 95% based on a prior study, to detect a difference in self-efficacy scale of 0.98. (12) Our study included adult participants aged 18 years or older residing in the Aseer Region, Saudi Arabia, with Internet access. To accurately categorize them into controlled and uncontrolled diabetes, we requested participants to upload their most recent HbA1C laboratory result conducted within the past 3 months. Responders who were unable to provide an accurate report of their latest laboratory HbA1C result were excluded. Additionally, we excluded participants with incomplete and inconsistent data from the analysis.

### 2.2 Data collection

Before actual data collection, data collectors were asked to collect five responses to assess the time needed to complete the questionnaire, the clarity of the language, and the response rate. The response rate was 84%, the time to complete the questionnaire ranged from 5 to 12 minutes and a few rewordings were made to improve the language of the questionnaire. The data of the pilot study was excluded from the final data set.

The study questionnaire was uploaded to Google Form and circulated through commonly used social media platforms (Instagram, Facebook, and Twitter). The study collected demographic information like age, sex, marital status, employment status, occupation, residence, nationality, education, health insurance, and monthly income. In the second section, we asked about the comorbid conditions (cardiovascular diseases, cerebrovascular diseases, and cancer). We asked about the type of diabetes (Type 1 diabetes mellitus or Type 2 diabetes mellitus). Furthermore, we collected data regarding glycemic control based on the latest HbA1c. Lastly, we asked about the disease’s duration, participants were categorized as follows: (less than 1 year, 1- 2 years, 3- 5 years, 6-10 years, and more than 10 years). (7).

To measure patient self-efficacy, we used the validated Arabic version of SEM6S. This questionnaire consists of six items, employing a 10-step Likert scale ranging from 1 (‘not at all confident’) to 10 (‘totally confident’). The maximum self-efficacy score achievable is 60. When two consecutive numbers are circled, code the lower number to reflect lower self-efficacy. The overall scale score was determined by calculating the mean score of the six items. We established the median score of all respondents as the cutoff point to categorize patients into those with or without self-efficacy. (13) If more than two items are missing, the scale should not be scored (7).

We measured the patient’s well-being using the validated Arabic version of the WHO well-being questionnaire. WHO-5 is a concise self-administered well-being measure that covers the past two weeks. Comprising five positively framed items, respondents rate their experiences on a 6-point Likert scale, ranging from 0 (at no time) to 5 (all the time). The raw scores were then transformed into a scale from 0 to 100, where lower scores signify poorer well-being. A wellbeing score equal to or less than 50 signals suboptimal mental health and prompts a need for additional exploration of potential symptoms of depression. A score of 28 or lower specifically points towards the presence of depressive symptoms (14, 15).

### 2.3 Data analysis

All collected data were numerically coded, processed using Microsoft Excel, and analyzed using Statistical Package for the Social Sciences (SPSS) version 27. Descriptive statistics were used to summarize the study variables, with percentages and frequencies for categorical data and mean and standard deviation for numerical data. The Z test was used to assess the association between SEM6S and demographic characteristics. To detect the association between glycemic control and independent variables, we performed the Chi-square test. We developed two binary logistic regression models; the first was used to identify the determinant of glycemic control. The second model was used to assess the determinants of well-being. Statistical significance was set at *P* < 0.05.

### 2.4 Ethical considerations

Participants were made fully aware of the research’s goals prior to the study’s start. Each participant was then asked for their written informed consent. The study was approved by the King Khalid University Research Ethics Committee (IRB ECM#2023-3105). The Helsinki Declaration’s tenets were strictly followed throughout the research procedure.

## 3 Results

The initial sample comprised 410 diabetic respondents. However, we excluded the responses of 68 patients who did not upload their Hb1C test results. Nearly two-thirds of the participants were male (67.25%), 59.0% were married, and their mean age was 43.17 ± 17.61 years. Furthermore, 36.55% had income exceeding 10,000 SAR, 76.32% were not employed in the healthcare sector, and 42.69% had a university degree or higher. Furthermore, a large proportion of participants reported having diabetic relatives (70.18%) and 92.69% were aware of their type of diabetes. Over two-thirds of respondents had glycated hemoglobin levels greater than 7% (61.40%), and 54.0% had other chronic conditions. For more detailed information, refer to **Table 1**.

**Table 1:** Study respondents’ characteristics.

Studied variables	Characteristic N = 342	N (%)
Gender	Female	112 (33%)
	Male	230 (67%)
Age (years)	<20	24 (7%)
	21 - 35	99 (29%)
	36 - 50	90 (26%)
	51 - 65	92 (27%)
	66+	36 (11%)
	Mean ± SD	43.17±17.61
Marital status	Divorced/widow	28 (8%)
	Married	202 (59%)
	Single	112 (33%)
Monthly income (SAR)	<5000	100 (30%)
	5000-10000	94 (28%)
	>10000	125 (38%)
Employment status	Unemployed	14 (4%)
	No	261 (76%)
	Yes	81 (24%)
Education	Illiterate	25 (7%)
	Reads and write	18 (5%)
	Primary/preparatory	26 (8%)
	Secondary	93 (27%)
	University and postgraduate	180 (53%)
Health Insurance	Health insurance private	53 (15%)
	Health insurance Public	63 (18%)
	No insurance	226 (66%)
Smoking	Non-smoker	289 (85%)
	Smoker	53 (15%)
Occupation	Private sector	38 (11%)
	Public sector	102 (30%)
	Retired	69 (20%)
	Unemployed	133 (39%)
Residence	City	271 (79%)
	Village	71 (21%)
Nationality	Not Saudi	12 (4%)
	Saudi	330 (96%)
Duration of diabetes mellitus	<1	37 (11%)
	1 to 2	42 (12%)
	3 to 5	95 (28%)
	6 to10	55 (16%)
	>10	113 (33%)
Type of diabetes mellitus	Don't know	25 (7%)
	Type one	109 (32%)
	Type two	208 (61%)
Having chronic diseases	No	102 (30%)
	Yes	240 (70%)
Hemoglobin A1C	<7	132 (39%)
	>=7	210 (61%)

**Figure 2:**
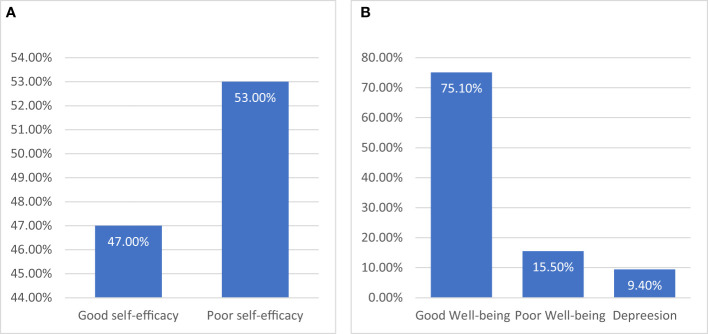
**(A)**, the distribution of study participants is presented, categorized according to self-efficacy, with the median score serving as the cutoff point. **(B)** illustrates the distribution of participants based on their well-being score. Individuals with scores of 28 or below were classified as experiencing depression, and those with scores of 50 or below were categorized as having poor well-being.

The distribution among respondents was relatively balanced, with 47.0% (n=161) exhibiting self-efficacy and 53.0% (n=181) lacking self-efficacy. **Figure 2A** Well-being was reported by more than three-quarters of the participants, accounting for 75.1%, while 24.9% reported poor (suboptimal) well-being, including 9.4% who were identified as experiencing depressive symptoms **Figure 2B**.

Around 40% of males and females were controlling their diseases, no significant difference in HbA1C test results and gender (*P* = 0.949). Marital status had no significant differences in any control of diabetes (*P* = 0.996). There was no significant difference in glycemic control according to income levels (*P* = 0.161). Similarly, there was no significant difference in glycemic control between health and non-health workers (*P* = 0.559). Individuals with higher education showed significantly better glycemic control (46.7%, *P* = 0.018). The study found a significant difference in glycemic control based on the type of social insurance. Patients with private insurance had the highest percentage of glycemic control (*P* = 0.002). There was also a significant difference in glycemic control based on occupation (*P* = 0.009), place of residence (cities and villages) (*P* = 0.030), presence of other chronic diseases (*P* = 0.034), and self-efficacy (*P* = 0.011). However, no significant association was found between smoking, nationality, type of diabetes, duration of disease, having diabetic relatives, and glycemic control (*P* = 1.000, 0.495, 0.200, 0.156, 1.000) **Table 2**.

**Table 2:** The distribution of respondents according to their characteristics and diabetes control.

label	levels	Controlled (n = 132)	Uncontrolled (n= 210)	P
**Gender**	Female	44 (39.3)	68 (60.7)	0.949
	Male	88 (38.3)	142 (61.7)	
**Age (years)**	< 20 years	5 (20.8)	19 (79.2)	0.066.
	21 – 35 years	44 (44.4)	55 (55.6)	
	36 – 50 years	41 (45.6)	49 (54.4)	
	51 – 65 years	32 (34.8)	60 (65.2)	
	66+ years	10 (27.8)	26 (72.2)	
**Marital status**	Divorced/Widowed	11 (39.3)	17 (60.7)	0.996
	Married	78 (38.6)	124 (61.4)	
	Single	43 (38.4)	69 (61.6)	
**Monthly income**	<5000 SAR	33 (33.0)	67 (67.0)	0.161
	>10000 SAR	58 (46.4)	67 (53.6)	
	5000-10000 SAR	35 (37.2)	59 (62.8)	
	Unemployed	4 (28.6)	10 (71.4)	
**Health worker**	No	98 (37.5)	163 (62.5)	0.559
	Yes	34 (42.0)	47 (58.0)	
**Education**	Illiterate	9 (36.0)	16 (64.0)	0.018*
	Reads and write	4 (22.2)	14 (77.8)	
	Primary/Preparatory	6 (23.1)	20 (76.9)	
	Secondary	29 (31.2)	64 (68.8)	
	University	84 (46.7)	96 (53.3)	
**Social insurance**	Health Insurance Private	31 (58.5)	22 (41.5)	0.002**
	Health insurance Public	27 (42.9)	36 (57.1)	
	No Insurance	74 (32.7)	152 (67.3)	
**Smoking**	Non-smoker	112 (38.8)	177 (61.2)	1.000
	Smoker	20 (37.7)	33 (62.3)	
**Occupation**	Private sector	24 (63.2)	14 (36.8)	0.009**
	Public sector	39 (38.2)	63 (61.8)	
	Retired	25 (36.2)	44 (63.8)	
	Unemployed	44 (33.1)	89 (66.9)	
**Place of residence**	City	113 (41.7)	158 (58.3)	0.030*
	Village	19 (26.8)	52 (73.2)	
**Nationality**	Not Saudi	3 (25.0)	9 (75.0)	0.495
	Saudi	129 (39.1)	201 (60.9)	
**Diabetes years**	<1	17 (45.9)	20 (54.1)	0.156
	>10	37 (32.7)	76 (67.3)	
	1 to 2	21 (50.0)	21 (50.0)	
	3 to 5	40 (42.1)	55 (57.9)	
	6 to10	17 (30.9)	38 (69.1)	
**Diabetes type**	Don’t know	6 (24.0)	19 (76.0)	0.200
	Type one	47 (43.1)	62 (56.9)	
	Type two	79 (38.0)	129 (62.0)	
**Diabatic relatives**	No	39 (38.2)	63 (61.8)	1.000
	Yes	93 (38.8)	147 (61.2)	
**Chronic diseases**	No	61 (33.2)	123 (66.8)	0.034*
	Yes	71 (44.9)	87 (55.1)	
**Self-efficacy**	Have efficacy	74 (46.0)	87 (54.0)	0.011*
	No efficacy	58 (32.0)	123 (68.0)	

*** p-value<0.001, ** p-value<0.01, * p-value<0.05.

There was a statistically significant difference in the mean self-efficacy scores between the controlled and uncontrolled diabetes groups (*P* = 0.031). similarly, the was a statistically significant difference in mean of well-being score across the studied group (*P* = 0.040) **Table 3**.

**Table 3:** The difference between patients with controlled and uncontrolled diabetes in wellbeing and self-efficacy.

Variables	Controlled	Uncontrolled	Z test	Mean Difference	P
Well-being	67.35± 21.22	60.93 ± 25.05	-2.96	-4.382	0.0031
Self-efficacy	40.86± 13.26	36.48± 13.26	-2.05	-4.667	0.040

**Table 4** shows the odds of having controlled diabetes in each category with respect to the reference category. The significant categories were self-efficacy score [OR=1.03(95%CI, 1.01-1.06, *P* = 0.002], having self-efficacy increased the odds of controlling diabetes than the reference category by 3%. Having other chronic diseases increased the odds of having control diabetes by 3.25 times compare with the reference category [OR = 3.25 (95%CI, 1.83-5.93), *P <*0.001]. Type 1 diabetes increased the odds of diabetes control [OR = 7.16, 95%CI, 1.96-30.71, *P* = 0.005]. Finally, being of Saudi nationality improved diabetes control [OR = 7.67, (95%CI, 1.37 – 53.84), *P* = 0.027]. However, the duration of diabetes between 6 and 10 years, and more than 10 years, was significantly associated with lower odds of diabetes control [OR = 0.33, 95% CI, 0.11-0.95), *P* = 0.043] and [OR = 0.32, 95% CI, 0.12-0.86), *P* = 0.026], respectively. Unemployment [OR = 0.19 (95% CI, 0.06-0.59), *P* = 0.005], working in the public sector [OR = 0.15 (95% CI, 0.05-0.44), *P* = 0.005], and being a smoker [OR = 0.44, 95% CI, 0.19-0.98, *P* = 0.048] also decreased the odds of having controlled diabetes. **Tables 4**, **5** shows the odds of having well-being in each category with respect to the reference category. The predictors of well-being were self-efficacy [OR=1.07 (95%CI, 1.04-1.09), *P* = 0.0001], education level (read and write), [OR=0.12 (95%CI,.02-.71), *P* = 0.021], and having public health insurance [OR=4.36 (95%CI, 1.36- 14.89), *P* = 0.015].

**Table 4:** Predictors of glycemic control among the study participants.

Predictors	OR	Lower CI	Upper CI	P value
(Intercept)	0.30	0.01	6.49	0.451
Self-efficacy (yes)	1.03	1.01	1.06	**0.002**
Age 21 - 35 years	2.06	0.57	8.48	0.289
Age 36 - 50 years	1.37	0.29	7.18	0.698
Age 51 - 65 years	0.75	0.14	4.43	0.750
Age +66 years	0.27	0.03	2.11	0.212
Gender (male)	0.91	0.47	1.78	0.789
Education (read and write)	0.32	0.05	1.93	0.230
Education (primary)	0.00	NA	6.27E+31	0.986
Education (preparatory)	0.29	0.04	1.79	0.185
Education (secondary)	0.33	0.06	1.68	0.182
Education (university)	0.43	0.08	2.18	0.308
Education (postgraduate)	0.42	0.07	2.49	0.344
Chronic diseases (Yes)	3.25	1.83	5.93	**0.0001**
Diabatic relatives (yes)	0.87	0.47	1.59	0.640
Diabetes (type one)	7.16	1.96	30.71	**0.005**
Diabetes (type two)	2.20	0.67	8.42	0.214
Diabetes duration (1 - 2 years)	0.86	0.28	2.66	0.797
Diabetes duration (3 - 5 years)	0.56	0.21	1.52	0.258
Diabetes duration (6- 10 years)	0.33	0.11	0.95	**0.043**
Diabetes duration (> 10 years)	0.32	0.12	0.86	**0.026**
Nationality (Saudi)	7.67	1.37	53.84	**0.027**
Place of residence (village)	0.56	0.26	1.16	0.123
Occupation (public sector)	0.15	0.05	0.44	**0.001**
Occupation (retired)	0.38	0.11	1.26	0.118
Occupation (unemployed)	0.19	0.06	0.59	**0.005**
Smoking (yes)	0.44	0.19	0.98	**0.048**
Health insurance (public)	0.55	0.21	1.44	0.228
Health insurance (no)	0.54	0.23	1.24	0.149
Healthcare worker (yes)	0.83	0.41	1.69	0.616
Monthly income 5000-10000	2.68	0.95	7.83	0.066
Monthly income (>10000 SAR)	1.20	0.48	2.99	0.694
Monthly income **(not fixed)**	0.89	0.17	4.05	0.881
Marital status (married)	0.49	0.14	1.66	0.253
Marital status (single)	0.38	0.08	1.75	0.218

Data in bold indicate significant findings.

**Table 5:** Predictors of well-being of diabetic patients among the study participants.

Predictors	Odds ratio	Lower CI	Upper CI	P
Self-efficacy (yes)	1.37	0.06	41.35	0.851
Age 21 - 35 years	1.07	1.04	1.09	**0.0001**
Age 36 - 50 years	0.98	0.26	3.57	0.980
Age 51 - 65 years	1.51	0.28	7.70	0.622
Age 66+ years	1.22	0.19	7.41	0.832
Age 66+ years	0.83	0.10	6.57	0.862
Gender (male)	1.54	0.74	3.24	0.250
Education (read and write)	0.12	0.02	0.71	**0.024**
Education (primary)	0.48	0.03	14.49	0.619
Education (preparatory)	0.23	0.03	1.81	0.168
Education (secondary)	0.24	0.03	1.38	0.123
Education (university)	0.26	0.04	1.51	0.147
Education (postgraduate)	0.42	0.05	3.26	0.409
Chronic diseases (yes)	0.71	0.38	1.34	0.294
Diabatic relatives (yes)	0.84	0.41	1.69	0.626
Diabetes (type one)	1.03	0.27	3.60	0.958
Diabetes (type two)	1.03	0.30	3.16	0.959
Diabetes duration (1 - 2 years)	2.78	0.76	11.28	0.132
Diabetes duration (3 - 5 years)	1.01	0.35	2.83	0.983
Diabetes duration (6- 10 years)	0.91	0.30	2.73	0.865
Diabetes duration (> 10 years)	1.72	0.62	4.69	0.287
Nationality (Saudi)	0.52	0.06	2.80	0.476
Place of residence (village)	1.05	0.49	2.34	0.895
Occupation (public sector)	0.54	0.14	1.90	0.354
Occupation (retired)	1.11	0.25	4.80	0.887
Occupation (unemployed)	0.52	0.14	1.82	0.323
Smoking (yes)	0.80	0.34	1.95	0.612
Health insurance (public)	4.36	1.36	14.89	**0.015**
Health insurance (no)	1.41	0.57	3.44	0.454
Healthcare worker (yes)	1.36	0.61	3.11	0.457
Monthly income 5000-10000	0.68	0.24	1.90	0.465
Monthly income (>10000 SAR)	0.44	0.13	1.39	0.166
Income (not fixed)	0.81	0.18	3.98	0.787
Marital status (married)	1.83	0.51	6.61	0.352
Marital status (single)	1.16	0.23	5.67	0.857

Reference Category: Self-efficacy: No, Age: < 20 years, Education: Illiterate, Chronic diseases: No, Diabatic relatives: No, Diabetes type: Type two, Diabetes duration: < 1 year, Nationality: Non-Saudi, Place of residence: Urban, Occupation: Private sector, Smoking: Nonsmoker, Health insurance: private insurance, Health worker: No, Income: < 5000 SAR/month, Marital status: widow/divorced, Gender: Female, Dependent variable well-being (Yes Vs. No). Data in bold indicate significant findings.

## 4 Discussion

This study focused on assessing how self-efficacy influences diabetes control, measured by evaluating HbA1c levels in the previous three months. Alongside this, we investigated the well-being of the participants, aiming to establish the predictive role of self-efficacy in determining well-being. By exploring the association between self-efficacy, diabetes control, and well-being, our goal was to contribute valuable insights that could inform strategies and interventions for improving the comprehensive health outcomes of individuals managing diabetes.

### 4.1 Main study findings

Approximately 25.0% of the respondents reported a poor well-being, 9.4% experienced depression, while 47.0% indicated self-efficacy. Factors that contributed to improved glycemic control included self-efficacy, the presence of chronic health conditions, being Saudi, and having Type 1 diabetes. On the contrary, longer durations of diabetes, working in the public sector, unemployment, and smoking were associated with lower odds of controlling diabetes. Regarding well-being, individuals with basic education (read and write) showed lower odds, whereas patients with higher self-efficacy and having public health insurance exhibited higher odd of well-being.

### 4.2 Interpretation of the findings

#### 4.2.1 Factors associated with glycemic control

We found that having diabetes for a duration longer than 6 years was significantly associated with worse glycemic control. In the same vein, Juarez et al. (16) Shamshirgaran et al. (17), and Mamo et al. (18) reported that glycemic control tends to worsen with the duration of diabetes. The longer duration of diabetes, especially uncontrolled diabetes, was found to be associated with mortality from other diseases as well. (19) The poor control of diabetes with increasing diseases duration could be attributed to a decrease in insulin secretion or an elevated level of insulin resistance among those patients (20).

Notably, individuals without comorbidities exhibit significantly better glycemic control, with an OR of 3.25 compared to those with comorbid conditions. In line with our findings, several studies showed a significant association between the number of chronic conditions and glycemic control. (21, 22) The observed association between multimorbidity and improved glycemic control could be attributed to more effective healthcare management for patients with multiple chronic conditions. In simple terms, the presence of comorbidities may open opportunities for a more comprehensive treatment approach, which may help to achieve the desired level of glycemic control. However, previous research has produced inconclusive results. For example, Haghighatpanah et al. (20) found that the presence of comorbidity was comparable in people with good and bad glycemic control. We found that the Saudi population has a higher probability of managing diabetes compared to other nationalities. This may be due to ongoing intervention programs that targets general population and medical students to raise their knowledge about diabetes and obesity (23, 24) and their complications (25).

Our findings indicated a significant association between smoking and poorer glycemic control. Consistently, data from the Swedish National Diabetes Registry covering the years 1996–2001 revealed that smokers exhibited higher mean HbA1c levels compared to non-smokers. (26) Similarly, the Fukuoka Diabetes Registry findings highlighted that Japanese men with Type 2 diabetes mellitus who smoked experienced a significant increase in mean HbA1c levels compared to non-smokers. (27) Further supporting this trend, in a study involving 10,551 men with diabetes in China, smoking was associated with an elevated risk of poor glycemic control. (28) In another Chinese study, male heavy smokers with Type 2 diabetes mellitus undergoing medical treatment exhibited a mean HbA1c increase of 0.38% compared to non-smokers. (29) These collective findings underscore the consistent association between smoking and compromised glycemic outcomes across diverse populations. Smoking can potentially impact glucose regulation directly by engaging various mechanisms, including the elevation of insulin resistance, reduction in insulin secretion, or impairment of pancreatic beta cell function (30, 31).

#### 4.2.2 Self-efficacy and glycemic control

Self-efficacy plays a significant role in the self-management of diabetes and serves as a predictor of its outcomes. In this study we found that self-efficacy was significantly associated with glycemic control in bivariate and multivariate analysis. In a similar vein, Dehghan et al. (32) revealed that self-efficacy played a noteworthy role by accounting for a considerable portion of the variability in diabetes self-care (11.4%) and influencing a significant part of the variance in behavioral intention related to diabetes self-care (31.3%). Sarkar et al. (33) found that with each 10% rise in self-efficacy score, patients demonstrated an increased probability of reporting adherence to an optimal diet, heightened involvement in exercise, and more frequent self-monitoring of blood glucose and foot care. Nevertheless, no notable association was detected with medication adherence. Moreover, a study involving 200 diabetic patients in Nigeria revealed that self-efficacy emerged as the most influential predictor associated with glycemic control, surpassing factors such as age, adherence to treatment, and engagement in physical exercise. (34) In a meta-analysis conducted by Jiang et al. (35), it was determined that educational interventions emphasizing self-efficacy are probable to reduce HbA1C levels, elevate self-efficacy, regulate self-management behaviors, improve knowledge, and ultimately contribute to an improved QoL for individuals with diabetes. Importantly, the association between self-efficacy and self-management remained consistent between different groups of race/ethnicity and levels of health literacy. (34, 36, 37). This finding addresses the importance of implementing programs to increase self-efficacy to improve glycemic control. On the contrary, Dehghan et al. (32) did not identify a significant association between self-efficacy and glycemic control among Iranians. In their study, the only significant predictor of glycemic control was the duration of the disease. Numerous studies have also indicated the absence of a link between self-efficacy and the management of metabolic syndrome (38, 39).

### 4.3 Well-being among diabetic patients

In this study, it was observed that the well-being of individuals with uncontrolled diabetes was significantly lower compared to those with controlled diabetes. Additionally, the study identified self-efficacy as a significant predictor of well-being, alongside the presence of health insurance and education. Similarly, Çalli and Kartal (34) found that self-efficacy was the strongest predictor of well-being. Psychological well-being indicators, such as optimism, positive affect, self-efficacy, and gratitude, have consistently been associated with better health outcomes in various medical conditions. These associations have been observed prospectively, irrespective of sociodemographic and medical factors. (33) Mental distress and depression seem to exert a significant influence on health behavior and medical outcomes, particularly among patients with diabetes (40, 41). For instance, distress is correlated with reduced treatment adherence (42), while depression is associated with compromised glucose control (43), end organs complications (43), and increased mortality (43–45).

### 4.4 Implication of the current study

This study has important implications for healthcare care, suggesting the need for comprehensive approaches to diabetes care. The prevalence of poor well-being highlights the importance of integrating mental health support into the management of diabetes. Factors that influence glycemic control, such as self-efficacy, age, occupation, and duration of diabetes, provide targeted areas for interventions. Tailoring education to individuals with higher odds of non-controlled diabetes and enhancing self-efficacy may improve diabetes outcomes.

### 4.5 Strengths and limitation

This study has several strengths that contribute to the validity of its findings. The extensive nature of data collection, which includes variables ranging from demographics to self-efficacy and well-being, provides a comprehensive understanding of the factors that influence diabetes management. Furthermore, the use of validated Arabic measurement tools, such as the SEM6S and the WHO-5 questionnaire, ensures the data’s reliability and validity. Furthermore, the exclusion of respondents who were unable to accurately report the HbA1C results reduces the study’s potential bias. Despite its strengths, the study has notable limitations that should be considered. The use of convenient and snowball sampling methods may introduce selection bias, potentially limiting the generalizability of findings to the Asser region’s larger diabetic population. Furthermore, while employing an online survey is convenient, it may inadvertently exclude individuals without internet access, potentially impacting the inclusivity and representativeness of the sample. However, it’s noteworthy that a substantial segment of the Saudi population actively uses social media platforms such as TikTok, Facebook, Telegram, Snapchat, and YouTube, which could help mitigate this bias (46). The cross-sectional design restricts the study to capturing a single point in time, preventing the establishment of causal relationships. Finally, we did not ask about dietary habits, treatment regimen, and complications like frequency of hypoglycemic and hyperglycemic attacks.

## 5 Conclusions

This study provides valuable insights into diabetes control, shedding light on both non-modifiable and modifiable factors influencing its management. Among these factors, self-efficacy emerges as a significant determinant. Demographic factors such as sex, marital status, and income did not show significant associations with glycemic control, but non-modifiable risk factors such as nationality and modifiable risk factors such as self-efficacy, occupation, and smoking emerged as a key factor in glycemic control. The distribution of reported self-efficacy is nearly balanced among diabetic patients. A noteworthy proportion report a suboptimal well-being, shedding light on the prevalent mental health challenges within diabetic patients. To address these findings, it is recommended to implement targeted educational interventions aimed at enhancing awareness of diabetes management. These interventions can be delivered through the community-based participation approach, a method that has been proven to be economically effective in diabetes management. (47) Additionally, the use of social media platforms could serve as another effective approach to providing such services. Moreover, psychosocial support programs should be integrated to improve self-efficacy, potentially improving disease control and mental well-being. Finally, inclusive healthcare policies are essential, considering factors like smoking, occupation, nationality, and the presence of other chronic conditions. Implementing these recommendations will enable healthcare practitioners and policymakers to design more effective and tailored interventions, ultimately enhancing diabetes control and well-being in the surveyed population.

## Data availability statement

The raw data supporting the conclusions of this article will be made available by the authors, without undue reservation.

## Ethics statement

The studies involving humans were approved by the Ethical Committee of King Khalid University, Abha, Saudi Arabia (IRB ECM#2023-3005). The studies were conducted in accordance with the local legislation and institutional requirements. The participants provided their written informed consent to participate in this study. Written informed consent was obtained from the individual(s) for the publication of any potentially identifiable images or data included in this article.

## Author contributions

AyA: Conceptualization, Data curation, Formal analysis, Methodology, Writing – original draft, Writing – review & editing, Funding acquisition, Supervision. FA-Q: Data curation, Writing – review & editing, Resources. SA: Resources, Investigation, Supervision, Writing – original draft. AhA: Investigation, Methodology, Software, Writing – review & editing. AMN: Data curation, Methodology, Supervision, Writing – original draft. AM: Methodology, Data curation, Supervision, Writing – review & editing. RA: Conceptualization, Investigation, Software, Writing – review & editing. AFA: Data curation, Methodology, Conceptualization, Writing – review & editing. ASA: Conceptualization, Investigation, Software, Writing – review & editing. HA: Conceptualization, Investigation, Software, Writing – original draft. ZSS: Conceptualization, Investigation, Writing – review & editing. AKA: Data curation, Methodology, Writing – original draft, Supervision. RG: Data curation, Methodology, Writing – original draft, Conceptualization, Formal analysis, Investigation, Software, Validation, Writing – review & editing.
